# Size reduction and performance improvement of a microstrip Wilkinson power divider using a hybrid design technique

**DOI:** 10.1038/s41598-021-87477-4

**Published:** 2021-04-08

**Authors:** Mohammad Behdad Jamshidi, Saeed Roshani, Jakub Talla, Sobhan Roshani, Zdenek Peroutka

**Affiliations:** 1 Research and Innovation Centre for Electrical Engineering (RICE), University of West Bohemia, Pilsen, Czech Republic; 2 Department of Power Electronics and Machines (KEV), University of West Bohemia, Pilsen, Czech Republic; 3 Department of Electrical Engineering, Kermanshah Branch, Islamic Azad University, Kermanshah, Iran

**Keywords:** Electrical and electronic engineering, Energy science and technology

## Abstract

In the design of a microstrip power divider, there are some important factors, including harmonic suppression, insertion loss, and size reduction, which affect the quality of the final product. Thus improving each of these factors contributes to a more efficient design. In this respect, a hybrid technique to reduce the size and improve the performance of a Wilkinson power divider (WPD) is introduced in this paper. The proposed method includes a typical series LC circuit, a miniaturizing inductor, and two transmission lines, which make an LC branch. Accordingly, two quarter-wavelength branches of the conventional WPD are replaced by two proposed LC branches. Not only does this modification lead to a 100% size reduction, an infinite number of harmonics suppression, and high-frequency selectivity theoretically, but it also results in a noticeable performance improvement practically compared to using quarter-wavelength branches in the conventional microstrip power dividers. The main important contributions of this technique are extreme size reduction and harmonic suppression for the implementation of a filtering power divider (FPD). Furthermore, by tuning the LC circuit, the arbitrary numbers of unwanted harmonics are blocked while the operating frequency, the stopband bandwidth, and the operating bandwidth are chosen optionally. The experimental result verifies the theoretical and simulated results of the proposed technique and demonstrates its potential for improving the performance and reducing the size of other similar microstrip components.

## Introduction

As modern communication systems have grown rapidly, the demands for microwave components with low energy loss, compact size, and filtering response have increased significantly. In this regard, wireless components should be integrated for the multiple function ability in embedded systems. For example, integrating a filter with a power divider will result in a compact size and low energy loss in the microwave components. Power dividers and couplers are two important microstrip passive components that can benefit from such modifications. Accordingly, they can have more appropriate harmonic suppression and higher performance than the conventional ones, leading to higher efficiency in aspects of the performance and energy^1,2^.

Power dividers play an important role in modern wireless communication systems^3^. Wilkinson power divider (WPD) is one of the most popular types of dividers. In fact, the WPD was first proposed by Ernest J. Wilkinson in 1960^4^. Since then it has widely used in modern communication systems, such as Doherty power amplifiers^5^, balanced power amplifiers^6,7^, push–pull power amplifiers^8^, antenna feed networks^9^, phase shifters^10^, and RF/microwave frontend systems^11^.

One of the disadvantages of the typical WPD is that the spurious harmonics can easily pass through the power divider paths and decrease its performance. Another disadvantage of the WPD is its large size, which is about approximately 0.0156 λ^2^. Recently, several approaches have been proposed and performed to solve these problems. For example, coupled lines^12^, open-ended stubs^13^, T-shaped and Π-shaped resonators^14^, and embedded filters^15^.

Open-ended stubs are usually used in the main quarter wavelength branches of the divider as studied in^13,16^ to reduce the size and suppress a few harmonics. Open-ended stubs can also be used at the input port to improve the input return loss parameter^17–19^. Applying open-ended stubs technique causes to provide desirable transmission zeros at the frequency response, which can suppress the desired harmonics and reduce the size^20^. However, the drawback of this method is to provide a single transmission zero. Hence, for obtaining a wide stopband several open-ended stubs should be applied^13^. Nevertheless, increasing the numbers of such stubs may contribute to the overall size and make the design more complicated.

Another technique, which has been used in several pieces of research, is the use of coupled lines^21,22^. Applying coupled lines in the divider structure can produce several transmission zeros and increase the operating bandwidth of the divider. Wang et al*.* utilized coupled lines in a WPD to obtain wideband and harmonic suppression^21^. However, the WPD designed in^21^ had a rather large size compared to the conventional divider. Furthermore, parallel-coupled lines to present a filtering power divider (FPD) with a filtering response were used in^22^. The frequency response of this divider was wideband with a filtering shape, but the size reduction was not achieved. Moreover, stepped impedance resonators to improve the performances of the dividers were applied^23,24^ and a dual-band filtering divider was introduced in^23^. In the presented divider in^21^, the stepped impedance resonators were applied to provide filtering response in operating frequencies, but the overall size of the divider was quietly large. In addition, the stepped impedance resonators for harmonic suppression in WPD were utilized in^24^. However, the obtained suppressing bandwidth was narrow and the designed WPD had a large size.

In fact, microstrip filters can suppress the desired frequency band; therefore, they can be embedded in the divider to make a filtering divider^25^. In this respect, substrate integrated waveguide (SIW) with embedded filters to design a filtering power divider were used in^15^, and multiple isolation resistors technique was also presented in^15^ in order to improve the divider isolation. Nonetheless, the overall size of the divider was very larger than the conventional divider.

Moreover, Chao and Li introduced a new technique to design an FPD, in which a resistor-inductor-capacitor isolation network was employed ^26^. While using lumped elements to design the suggested network in ^26^ was considered analytically, no lumped elements, such as inductors or capacitors were applied practically. Indeed, only microstrip lines were utilized in the fabrication stage. Disregarding the implementation of the introduced FDP in ^26^, the parallel inductor and capacitor were placed between output ports to achieve filtering response, but the suppression bandwidth was not wide enough and only the second harmonic was suppressed. Due to inserting the parallel inductor and capacitor between output ports, the high suppression band was not achieved in ^26^. In other words, this mechanism could only improve the isolation between the output ports (S_32_) and was not able to amend the filtering response (S_21_) effectively. Although it was claimed that “a miniaturized divider is obtained” in ^26^, the divider size is even larger than the first WPD made by E.J Wilkinson in 1960.

In another work^27^, A. Chen et al*.* added two capacitors in parallel with the transmission lines in a WPD to provide a new zero-reflection frequency, which resulted in a wider bandwidth compared to the conventional WPD. Although they designed a tunable WPD with a broad operating band, neither remarkable size reduction nor noteworthy harmonics suppression was achieved using the capacitors, which were added to the proposed WPD in^27^. According to the results of^27^, only the second harmonic was suppressed, which is not a desirable performance compared to some pieces of research^13,14,16,19,21–23^, and more importantly, size reduction was only around 23% that is not impressive for a WPD. Besides, the length of the long quarter wavelength branches, in the designed divider in^27^, was not reduced, but these branches were bent to achieve a limited size reduction.

On the other hand, combinations of different structures have been used in order to improve the characteristics of WPDs in some methods^23,28,29^. For instance, in^28^ the stepped impedance and open-ended stubs were combined, whereas stepped impedance, open-ended stubs, and coupled lines were employed to design a filtering divider in^29^. In addition, lumped elements, coupled lines, and open-ended stubs techniques were gathered to design a harmonic suppressed divider with a wide operating band in^29^.

As reviewed above, limited improvement in harmonic suppression and some restrictions in reducing the size should be taken into account as two important issues in the design of WPDs in all of the aforementioned methods. In the present paper, a novel hybrid method is proposed using LC branches in both paths of the divider to improve the performance of the WPD. In this regard, an infinite number of harmonics suppression and 100% size reduction can be achieved theoretically. Although, in practice, it is impossible to reach 100% size reduction and the infinite number of harmonics suppression, the extreme size reduction and large numbers of harmonic suppression are obtained, which has not been achieved in any divider so far. Most importantly, an analytical and flexible design method is offered, in which all of the key specifications of the WPDs can be chosen arbitrarily by the designers or manufacturers based on consumer tastes. These specifications include size reduction percentage, the number of harmonics suppression, operating frequency, and operating bandwidth.

The paper organization is demonstrated as follows. In “Typical structure of a microstrip Wilkinson power divider”, the conventional WPD and its fundamentals are described. In “Proposed WPD structure”, it is explained that how the proposed LC branches structure is applied in a typical WPD. The analytical equations of the introduced divider are then illustrated in “Analyses of the proposed WPD”. In “Design Examples at 2.4 GHz” and “Design examples at 0.8 GHz”, two design examples at 2.4 GHz and two design examples at 0.8 GHz are presented to verify the performance of the proposed structure in different frequencies and different dimensions. In “Measurement results”, the analytical and simulation results of the proposed structure are verified using experimental results. Finally, in the last “Conclusions”, the summary of the proposed method, its potentials, and conclusions are described.

## Typical structure of a microstrip Wilkinson power divider

**Figure 1:**
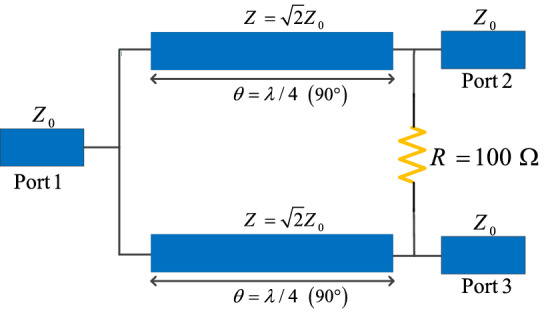
The arrangement of microstrip transmission lines to design a typical WPD.

**Figure 2:**
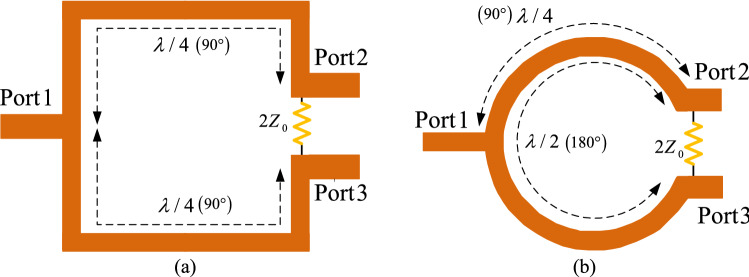
The arrangements of microstrip transmission lines in classic WPD layout; (**a**) square-shaped WPD layout; (**b**) circular-shaped WPD layout.

In this section, the fundamentals and basics of a typical WPD are described. In such components, quarter-wavelength branches are responsible to suppress the unwanted harmonics and passing the main frequency. Figure 1 illustrates a classic WPD with two quarter-wavelength branches and a resistor between output ports. One of the most challenging drawbacks with such structures is to have a large size due to utilizing the large quarter-wavelength branches, which are not favorable in modern wireless systems. Another important disadvantage, which can be mentioned for this type of design, is to suffer from the existence of spurious harmonics because the typical quarter-wavelength branches cannot provide a suppression band for the divider. Nevertheless, the WPD layout structure plays an important role in determining the overall size of WPDs. Generally, two common forms of the WPD have been utilized in the design of WPDs, namely squared-shape and circular-shaped, which are depicted in Fig. 2a and b, respectively. As you can see in Fig. 2a, the overall size of the square-shaped WPD, is 0.125 λ × 0.125 λ, which is equal to 0.0156 λ^2^ while the overall size for circular one is 0.0198 λ^2^, indicating this point that the circular type of the WPD is typically larger than the square-shaped one. It should be noted that in both square-shaped and circular-shaped WPDs, the size of the resistor is neglected in calculation of the total size theoretically. Regardless of this difference, the size of WPD and many other microstrip components significantly depend on their quarter-wavelength branches, such as branch-line couplers, rat-race couplers, and several types of discrete power amplifiers. The proposed structure will be discussed in the next section.

## Proposed WPD structure

As is mentioned, the typical WPD suffers from large size and spurious harmonics existence. Therefore, by modifying the transmission lines used in the WPD that are more responsible in increasing the size, not only will the size of components made by this technology be far more compact, but it can also lead to increasing the efficiency. Overall, to reach these objectives, some important parameters like selectivity and suppressing the unwanted harmonics should be considered. In this regard, a novel hybrid solution to improve the size and performance of a WPD has been presented here. In addition, the proposed method can be used in a wide range of microstrip components using transmission lines.

### Proposed LC branch lines

**Figure 3:**
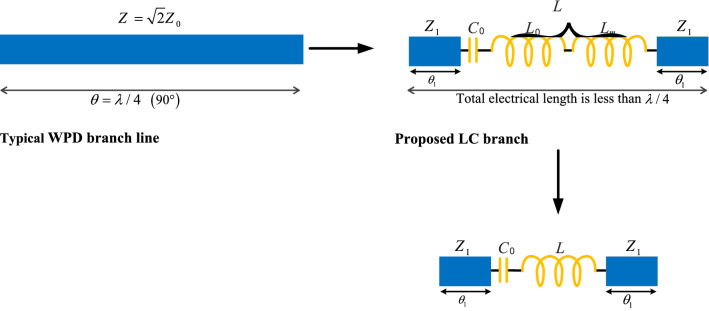
The main idea of replacing the microstrip lines with the proposed LC branches.

The proposed method includes replacing long quarter wave length transmission lines, which are used in WPDs or any other microwave components, such as filters, diplexers, matching networks, couplers, and power amplifiers, with the proposed compact LC branches. Figure 3 demonstrates the proposed hybrid approach using microstrip lines and LC branches in a microwave component. As is observed in Fig. 3, microstrip branch lines of the conventional WPD are replaced by the proposed LC branch lines. The LC branch lines include a resonance capacitor (C_0_), a resonance inductor (L_0_), a miniaturizing inductor (L_m_), and two transmission lines (Z_1_, θ_1_). The resonance capacitor and inductor, which form a series LC circuit, should be tuned at the desired operating frequency (*f*_0_). The series LC circuit is shorted at *f*_0_ while it is opened at other frequencies, resulting in the bandpass response of the divider. Therefore, the LC branch structure can be used to reduce the desired harmonics.

**Figure 4:**
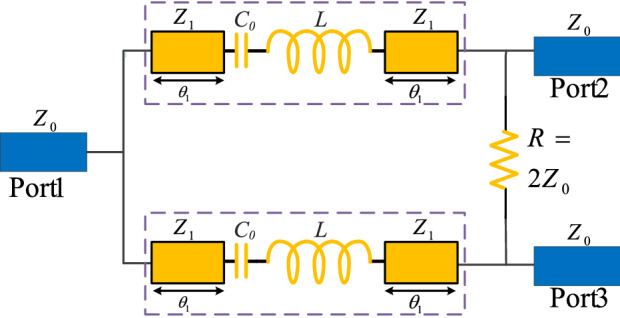
The proposed structure of the WPD with the presented LC branches.

Furthermore, the size of each microstrip branch line significantly decreases from a large size to a very small size that will be discussed in the next sections. In fact, the miniaturizing inductor results in reducing the LC branch length. The inductances of L_0_ and L_m_ are in series and can be considered as the inductance of L in practice. After applying the proposed LC branches, a new structure will be obtained for the WPD. The proposed structure of the WPD with the presented LC branches is shown in Fig. 4.

## Analyses of the proposed WPD

The structure of the proposed WPD is analyzed using the ABCD matrix. The ABCD matrix of the proposed LC branch has to be equaled to the ABCD matrix of a conventional quarter wavelength (QWL) branch. Therefore, the obtained equation is written in Eq. (1)

1 $$ {\text{M}}_{{1}} {{ \times }}\;{\text{M}}_{{{\text{LCB}}}} {{ \times }}\;{\text{M}}_{{1}} { = }\;{\text{M}}_{{{\text{QWL}}}} $$

where, in M_1_, M_LCB_, and M_QWL_ are ABCD matrices of the transmission line (Z_1_, θ_1_), LC branch (LCB), and quarter wavelength line ($\sqrt{2}$ Z_0_, λ/4), respectively. In addition, the values of M_1_, M_LCB_, and M_QWL_ matrices are defined in Eq. (2).

2 $$ \begin{gathered} {\text{M}}_{{1}} \; = \left[ {\begin{array}{*{20}c} {\cos \left( {\theta_{1} } \right)} & {jZ_{1} \sin \left( {\theta_{1} } \right)} \\ {jY_{1} \sin \left( {\theta_{1} } \right)} & {\cos \left( {\theta_{1} } \right)} \\ \end{array} } \right],\;\; \hfill \\ \hfill \\ {\text{M}}_{{{\text{LCB}}}} \; = \left[ {\begin{array}{*{20}c} 1 & {jL_{m} \omega + jL_{0} \omega - j/\left( {C_{0} \omega } \right)} \\ 0 & 1 \\ \end{array} } \right], \hfill \\ \hfill \\ {\text{M}}_{{{\text{QWL}}}} \; = \left[ {\begin{array}{*{20}c} 0 & {j\sqrt 2 Z_{0} } \\ {j/\sqrt 2 Z_{0} } & 0 \\ \end{array} } \right] \hfill \\ \end{gathered} $$

As mentioned before, the series L_0_C_0_ is tuned at the main frequency (*f*_0_), which is shorted at the main frequency. Hence, if it is assumed to perform the analyses in the main frequency, the LC branch matrix can be simplified as written in Eq. (3).

3 $$ {\text{M}}_{{{\text{LCB}}}} \; = \left[ {\begin{array}{*{20}c} 1 & {jL_{m} \omega } \\ 0 & 1 \\ \end{array} } \right] $$

By solving Eq. (1), three independent equations are obtained as follows

4 $$ 2Z_{1} = L_{m} \omega_{0} \tan \left( {2\theta_{1} } \right) $$

5 $$ \sqrt 2 = \frac{{Z_{1} }}{{Z_{0} }}\sin \left( {2\theta_{1} } \right) + \frac{{L_{m} \omega_{0} }}{{2Z_{0} }} + \frac{{L_{m} \omega_{0} }}{{2Z_{0} }}\cos \left( {2\theta_{1} } \right) $$

6 $$ \frac{1}{\sqrt 2 } = \frac{{Z_{0} }}{{Z_{1} }}\sin \left( {2\theta_{1} } \right) - \frac{{Z_{0} L_{m} \omega_{0} }}{{2Z_{1}^{2} }} + \frac{{Z_{0} L_{m} \omega_{0} }}{{2Z_{1}^{2} }}\cos \left( {2\theta_{1} } \right) $$

By comparing Eqs. (5) and (6), the following equation is achieved.

7 $$ 2 - \frac{{Z_{1}^{2} }}{{Z_{0}^{2} }} = \frac{{\sqrt 2 L_{m} \omega_{0} }}{{Z_{0} }} $$

By solving Eq. (7), which is a second-order equation, the normalized value of Z_1_ can be calculated as written in Eq. (8).

8 $$ \frac{{Z_{1} }}{{Z_{0} }} = \frac{{ - \sqrt 2 + \sqrt {2 + \tan \left( {2\theta_{1} } \right)^{2} } }}{{\tan \left( {2\theta } \right)}} $$

**Figure 5:**
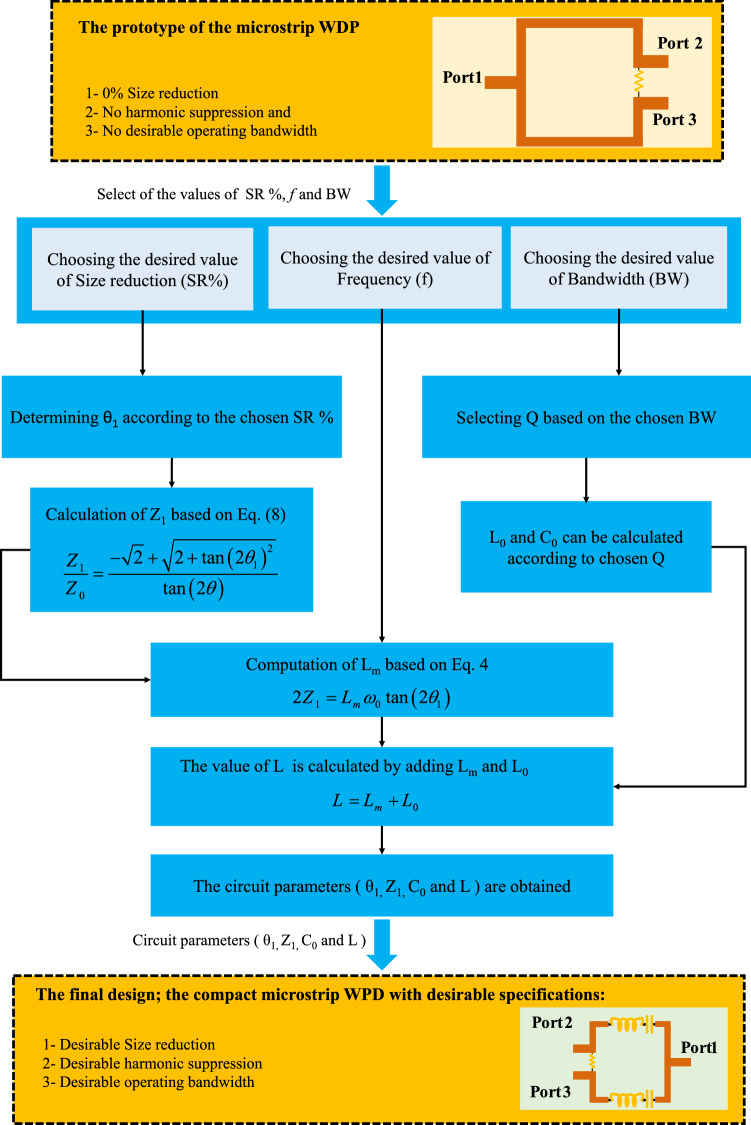
The process of design and solving the equations of a WPD based on the proposed technique. First, the conventional WPD with 0% size reduction, no harmonic suppression and no bandwidth tuning ability is considered. The desired values of SR%, operating frequency, and bandwidth are then assumed arbitrarily. Next, with the use of the proposed analyses, the equations are solved and the desired circuit parameters (θ1, Z_1_, C_0_, and L) are achieved. By applying these circuit parameters, and the proposed structure to the typical WPD, the new compact divider with desirable SR%, extreme harmonic suppression, and desirable operating bandwidth is obtained.

Figure 5 demonstrates the proposed approach, including the prototype of a microstrip WPD and the suggested method to solve the equations in order to obtain circuit parameters. As it can be seen in this figure, in the first stage, the prototype of the understudied component is indicated. In the second stage, the values of the key parameters for the component, such as the amount of size reduction (SR%), operating frequency (*f*), and operating bandwidth are arbitrarily determined. Next, θ_1_ is computed based on the selected SR% in the previous stage. In the third stage, Z_1_ is calculated using Eq. (8). The calculated circuit values in the third stage are listed in Table 1 at the operating frequency of 2.4 GHz and 0.8 GHz. In the fourth stage, the value of L_m_ is obtained by using Eq. (4). As is observed, the value of Z_1_ is independent of the operating frequency, while the value of L_m_ depends on the operating frequency. By adding L_0_ and L_m_, the total value of L is achievable in the fifth stage. The values of L_0_ and C_0_ are achieved based on values of Q (quality factor) being available in Tables 2 and 3 at the operating frequency of 2.4 GHz and 0.8 GHz. Besides, the value of Q is obtained through bandwidth, which will be more discussed at the next Sections. Finally, the new proposed divider with desirable parameters based on (θ_1_, Z_1_, C_0_, and L) can be achieved.

**Table 1:** The calculated circuit values of the divider for the desired values of the size reduction in two operating frequencies of 2.4 GHz and 0.8 GHz.

Total electrical length of branch 2θ_1_ (°)	θ_1_ (°)	Z_1_ (Ω)	L_m_ (nH) @2.4 GHz	L_m_ (nH) @0.8 GHz	Maximum size (λ_g_^2^) reduction of divider (%)
90° (0.25 λ)	45° (0.12 λ)	70.7	0	0	0
80° (0.22 λ)	40˚ (0.11 λ)	59.3	1.4	4.2	21
70˚ (0.19 λ	35° (0.10 λ)	49.5	2.4	7.2	39.5
60° (0.17 λ)	30° (0.08 λ)	40.8	3.1	9.4	55.5
50° (0.14 λ)	25° (0.07 λ)	33.0	3.7	11	69.1
40° (0.11 λ)	20˚ (0.05 λ)	25.7	4.0	12.2	80.2
30° (0.08 λ)	15°° (0.04 λ)	18.9	4.3	13	88.9
20° (0.05 λ)	10° (0.03 λ)	12.4	4.5	13.6	95
10° (0.03 λ)	5° (0.01 λ)	6.2	4.6	14	98.7

**Table 2:** Values of L_0_, C_0_, quality factor (Q), and operating bandwidth (BW) at 2.4 GHz operation frequency for the first and second design examples.

C_0_ (pF) 55.5% SR	L_0_ (nH) 55.5% SR	L_m_ (nH) 55.5% SR	L (nH) 55.5% SR	BW (MHz) 55.5% SR	Q 55.5% SR
0.2	22	3.1	25.1	600	4
0.5	8.8	3.1	11.9	1280	1.9
0.8	5.5	3.1	8.6	1830	1.3
1	4.4	3.1	7.5	2130	1.1

C_0_ (pF) 69.1% SR	L_0_ (nH) 69.1% SR	L_m_ (nH) 69.1% SR	L (nH) 69.1% SR	BW (MHz) 69.1% SR	Q 69.1% SR
0.2	22	3.7	25.7	500	4.8
0.5	8.8	3.7	12.5	1200	2
0.8	5.5	3.7	9.2	1650	1.4
1	4.4	3.7	8.1	1950	1.2

**Table 3:** Values of L_0_, C_0_, quality factor (Q) and operating bandwidth (BW) at 0.8 GHz operation frequency for the third and fourth design examples.

C_0_ (pF) 69.1% SR	L_0_ (nH) 69.1% SR	L_m_ (nH) 69.1% SR	L (nH) 69.1% SR	BW (MHz) 69.1% SR	Q 69.1% SR
0.2	198	11	209	75	10.7
0.5	79.2	11	90.2	150	5.3
0.8	49.5	11	60.5	230	3.5
1	39.6	11	50.6	300	2.7

C_0_ (pF) 80.2% SR	L_0_ (nH) 80.2% SR	L_m_ (nH) 80.2% SR	L (nH) 80.2% SR	BW (MHz) 80.2% SR	Q 80.2% SR
0.2	198	12.2	210.2	70	11.4
0.5	79.2	12.2	91.4	140	5.7
0.8	49.5	12.2	61.7	200	4
1	39.6	12.2	51.8	260	3

**Figure 6:**
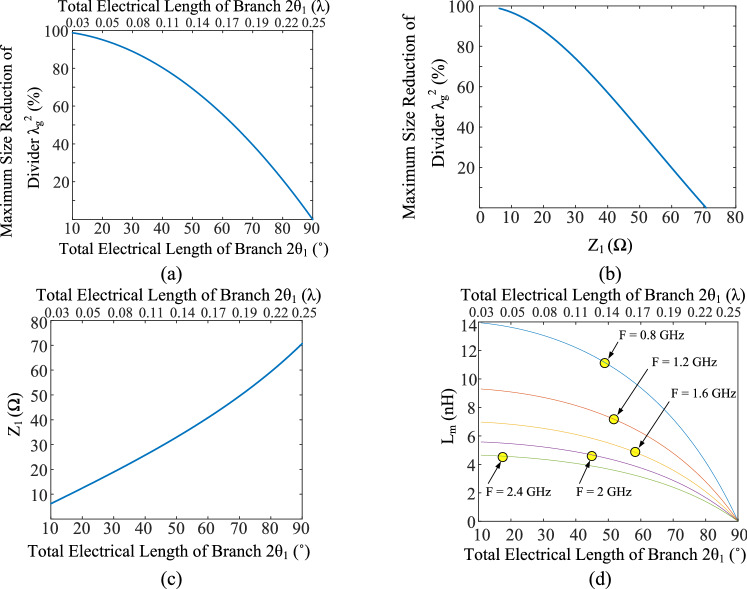
The calculated values of (**a**) maximum size reduction of divider versus total electrical length of branch and (**b**) versus value of Z_1_ (Ω). (**c**) The calculated values of Z_1_ (Ω) and (**d**) L_m_ (nH) versus total electrical length of branch. This Figure is illustrated by calculating the circuit parameters using proposed analyses, which can be followed in Eqs. (1)–(8) and Fig. 5.

The design process of the proposed technique is described in Fig. 5. In addition, some design examples for circuit parameters values are calculated and shown in Table. 1. Moreover, the relation between calculated circuit parameters from the analysis is shown in Fig. 6 for more clarification. The calculated values of divider maximum size reduction versus total electrical length of the branch and versus the value of Z_1_ (Ω) are demonstrated in Fig. 6a and b. The word "maximum" is used for maximum size reduction because the L and C component elements occupy a small size practically, which is not considered in the theory design. As mentioned above, the main branch line length of the conventional WPD is λ/4, while the proposed LC branch length is considered 2θ_1_. Therefore, the maximum size reduction is calculated according to the ratio of the size of the square shape WPD with the proposed LC branches to the size of the conventional square shape WPD with typical λ/4 branches. Figure 6b illustrates the lower size of the divider which is corresponding with the lower value of Z_1_, leading to a wider transmission line. Moreover, the calculated values of Z_1 _(Ω) versus the total electrical length of the branch are depicted in Fig. 6c. As can be seen in Fig. 6c, the lower size of the divider is corresponding with the wider transmission line. Besides that, Fig. 6d shows the calculated inductance L_m_ values versus maximum size reduction at different frequencies. According to this figure, the higher values of L_m_ are needed for higher size reduction and lower operating frequencies. Therefore, the values of Z_1_ are independent of the operating frequency, while the value of L_m_ depends the on operating frequency. Additionally, from both analyses and Fig. 6, it can be concluded that any value of maximum size reduction can be theoretically achieved using the proposed LC branches.

In the final design, in addition to L_m_, resonant inductor (L_0_) and capacitor (C_0_) will be applied to form the LC branch. The series LC circuit should be tuned at the desired operating frequency. For a better explanation of the design procedures, four design examples are introduced, two of them are at 2.4 GHz and two others are at 0.8 GHz. These design examples are comprehensively described in the next section.

## Design examples at 2.4 GHz

To verify the analytical results, two design examples at the operating frequency of 2.4 GHz are introduced in this section.

### Calculating the circuit parameters and circuit simulation results for the first and second design examples

**Figure 7:**
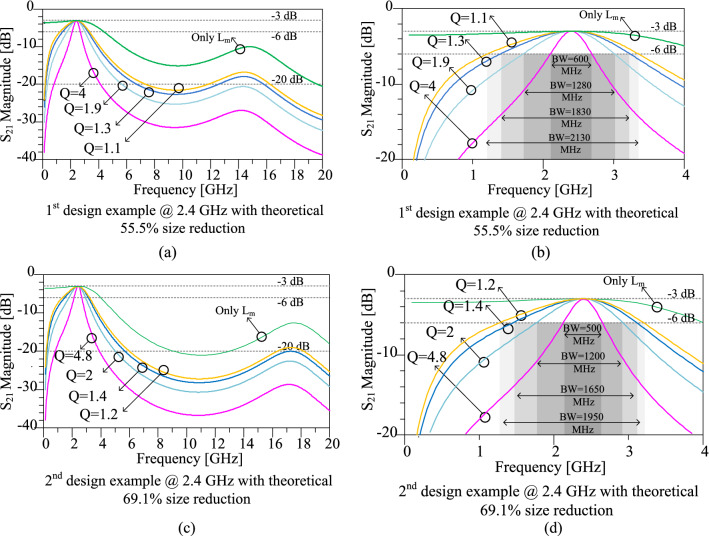
Results of the circuit simulations scattering parameter S_21_ of the first and second design examples. (**a**) First design example with theoretical 55.5% size reduction in the wide frequency range. (**b**) First design example with theoretical 55.5% size reduction at near operating frequency range. (**c**) Second design example with theoretical 69.1% size reduction in the wide frequency range. (**d**) Second design example with theoretical 69.1% size reduction at near operating frequency range. Effects of different quality factors of the series LC circuit on the simulated frequency responses can be seen in this figure. The operating frequency of 2.4 GHz is considered for first and second design examples.

Some examples for circuit parameter values with different size reductions are calculated and shown in Table. 1. The size reduction values of 55.5% and 69.1% are chosen for the first and second design examples, respectively, both at 2.4 GHz. The circuit parameters for the first and second design examples can be determined according to the assumed values of size reduction and operating frequency. From Table 1, the width (Z_1_) and length (θ_1_) dimensions of LC branches transmission lines are 40.8 Ω and 30˚ for 55.5% size reduction, while they are 33 Ω and 25° for 69.1% size reduction, respectively. The values of L_m_ are calculated equal to 3.1 nH and 3.7 nH for 55.5% and 69.1% size reduction values, respectively. In the next step, the series L_0_C_0_ circuit should be tuned at the operating frequency. However, the quality factor (Q) of the series L_0_C_0_ circuit can be tuned by changing the L_0_ and C_0_ values. The results of the circuit simulations for the first and second design examples at 2.4 GHz are illustrated in Fig. 7. Accordingly, the effects of the series L_0_C_0_ circuit with different quality factors on the simulated frequency responses of the presented design examples are shown in this figure. As can be seen in Fig. 7, by increasing the quality factor, the bandwidth decreases, whereas the stopband increases. In other words, by increasing the quality factor more harmonic suppression can be achieved. Therefore, the arbitrary bandwidth can be achieved by tuning the quality factor in LC branches. In the case indicated with “only L_m_” in Fig. 7, the L_m_ is only considered in the LC branch, while the resonant inductor (L_0_) and capacitor (C_0_) are not applied. This situation can also be assumed as a series resonant circuit with a small quality factor.

In the next step, the values of L_0_ and C_0_ should be determined according to the desired harmonic suppression and operating bandwidth of the WPD. Different values of L_0_ and C_0_ combinations are considered at 2.4 GHz operating frequency in Table 2. Then, the corresponding values of quality factors and operating bandwidths are extracted, according to the obtained results from circuit simulations of first and second design examples.

### Layouts and electromagnetic (EM) simulation results for the first and second design examples

**Figure 8:**
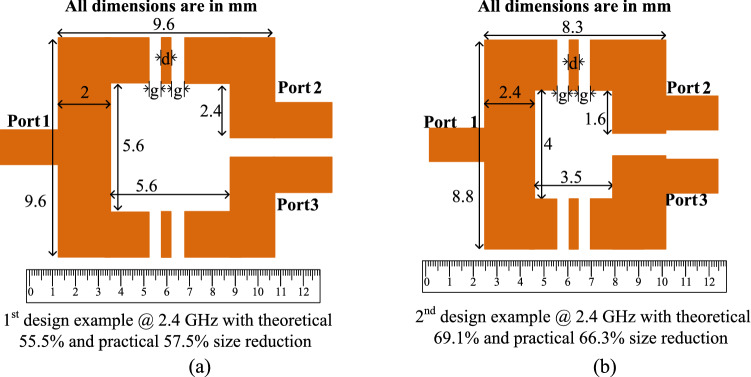
Layouts of the prototype dividers at 2.4 GHz for the (**a**) first design example with theoretical 55.5% size reduction and (**b**) second design example with theoretical 69.1% size reduction (All dimensions are in mm).

After calculating the circuit parameters and choosing the desired size reduction for the first and second design examples, which are explained in the previous “Calculating the circuit parameters and circuit simulation results for the first and second design examples”, the layouts of these two design examples can be determined. The considered substrate for the layout of the design includes an RT Duroid 5880 substrate with a thickness of 0.508 mm and ε_r_ = 2.2. The layouts of the prototype dividers at 2.4 GHz for the first and second design examples are depicted in Fig. 8. According to this Figure, the total size of the first and second design examples are 9.6 mm × 9.6 mm (0.1 λ_g_ × 0.1 λ_g_), and 8.3 mm × 8.8 mm (0.091 λ_g_ × 0.09 6λ_g_), respectively. To calculate the practical size reduction of the designed examples, a layout of the conventional squared shaped WPD at 2.4 GHz is designed, which the overall size of the conventional divider is obtained as 15.6 mm × 13.9 mm (0.17 λ_g_ × 0.15 λ_g_). Therefore, the practical size reduction of 57.5% and 66.3% are reached for the first and second design examples. Since some spaces should be considered for lumped elements in practice, there is a difference between theoretical and practical size reduction. Moreover, folding and bending the microstrip lines affect the theoretical values. In both first and second examples, the lengths of gaps (g) and intermediate transmission line between the gaps (d), which are used to solder the L and C components, are g = 0.6 mm and d = 0.5 mm, respectively.

**Figure 9:**
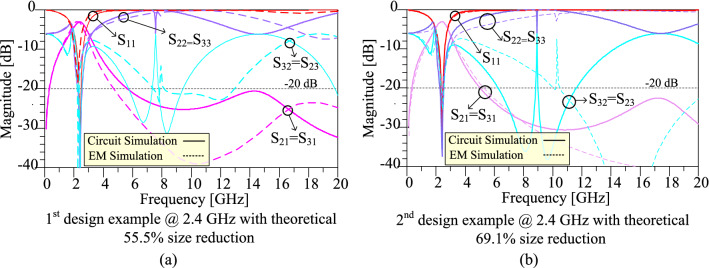
Frequency responses of the prototype dividers at 2.4 GHz for the (**a**) first design example with theoretical 55.5% size reduction and (**b**) second design example with theoretical 69.1% size reduction.

The frequency responses of the prototype dividers at 2.4 GHz for the first and second design examples are depicted in Fig. 9. The circuit simulation and electromagnetic (EM) simulation are compared in this Figure. As is observed, the obtained EM simulation results verify the circuit simulation results. According to the EM simulation results, for both first and second design examples, an appropriate suppression band is obtained, which can suppress 2nd up to 8th spurious harmonics. In addition, for both first and second design examples, insertion loss is below 0.1 dB in the operating frequency, and isolation between output ports is better than 35 dB in the operating frequency. The information about the EM simulation results for the first and second design examples is listed in Table 4 in "Measurement results" section.

**Table 4:** A comparison between the proposed divider results versus the previous approaches.

References		*f*_0_ (GHz)	HS	SR (%)		IL (dB)	Isolation (dB)	Methods	DC	DF
^15^		2.3	2nd	Very larger than conventional		1.2	20	SIW	High	Low
		3.5				1.6				
^16^		1	2nd 3rd	Larger than conventional		1.1	20	Open-ended stubs	Low	No
^26^		1	2nd	Larger than conventional		0.85	20	RLC isolation circuit	Medium	Low
^27^		0.75–2	2nd	23%		N/A	15	Parallel capacitances	Medium	Medium
^23^		2.0	2nd up to 6th	60%		0.28	14.4	Slow wave transmission lines (TLs), couple lines	High	No
		2.52								
		3.06								
^29^		1.99	2nd	Larger than conventional		0.74	20	Open-ended stubs, Coupled lines	Medium	No
The Proposed Technique	DEX1; Sim	2.4	2nd up to 8th	57.5%		0.1	40	LC branches	Low	High
	DEX2; Sim	2.4	2nd up to 8th	66.3%		0.1	35	LC branches	Low	High
	DEX3; Sim	0.8	2ndup to25th	70.5%		0.1	40	LC branches	Low	High
	DEX4; Sim	0.8	2nd up to 25th	82.8%		0.1	38	LC branches	Low	High
	DEX4; Mes	0.8	2nd up to 25th*	82.8%		0.3	15	LC branches	Low	High

*f*_0_ Operating Frequency, *HS* Harmonic Suppression, *SR* Size Reduction percent, *IL* Insertion Loss, *DC* Design Complexity, *DF* Design Flexibility, *DEX* Design Example, *Sim.* Simulation, *Mes.* Measurement.

*The 2nd, 3rd, 4th, 5th, 6th, 7th, 8th, 10th, 17th, 18th, 19th, 20th, 21th, 22th, 23th, 24th, and 25th harmonics are suppressed with suppression levels of more than 15 dB according to the measured results in the fabricated divider.

## Design examples at 0.8 GHz

Two design examples at the operating frequency of 0.8 GHz (third and fourth design examples) are introduced in this section to verify the analytical results at different frequencies.

### Calculating the circuit parameters and circuit simulation results for the third and fourth design examples

**Figure 10:**
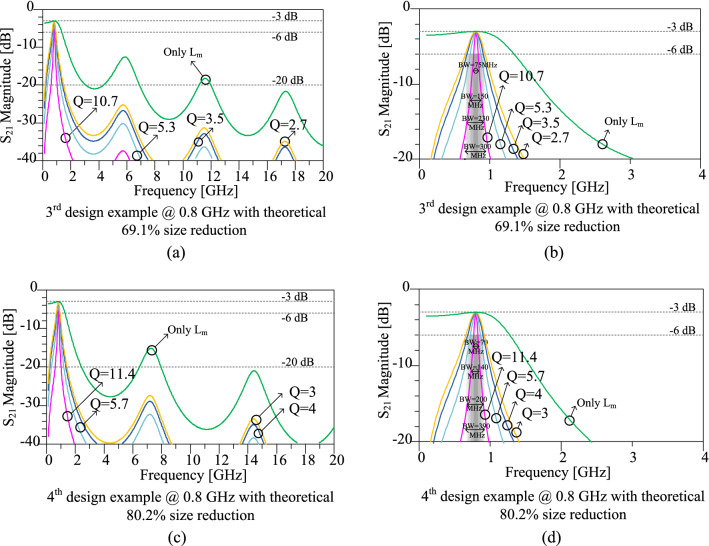
Results of the circuit simulations scattering parameter S_21_ of the third and fourth design examples. (**a**) Third design example with theoretical 69.1% size reduction in the wide frequency range. (**b**) Third design example with theoretical 69.1% size reduction at just around operating frequency range. (**c**) Fourth design example with theoretical 80.2% size reduction in the wide frequency range. (**d**) Fourth design example with theoretical 80.2% size reduction at about operating frequency range. Effects of different quality factors of the series LC circuit on the simulated frequency responses can be seen in this figure. The operating frequency of 0.8 GHz is calculated for the third and fourth design examples.

According to Table1, the size reduction values of 69.1% and 80.2% are chosen for the third and fourth design examples, respectively both at 0.8 GHz. The circuit parameters for the third and fourth design examples can be determined with the use of the assumed values of size reduction and operating frequency. As information is given in Table 1, the values of L_m_ should be equal to 8.8 nH and 9.8 nH for 69.1% and 80.2% size reduction values, respectively. The next steps of the design procedures are done similarly to the first and second design examples, which are explained in “Calculating the circuit parameters and circuit simulation results for the first and second design examples”. The results of the circuit simulations for the third and fourth design examples at 0.8 GHz are illustrated in Fig. 10. In addition, the effects of the series L_0_C_0_ circuit with different quality factors on the simulated frequency responses of the presented design examples are demonstrated in this figure. Hence, by increasing the quality factor, the bandwidth decreases while the stopband increases. In other words, by increasing the quality factor more harmonics can be suppressed.

In the next step, the values of L_0_ and C_0_ should be determined based on the desired harmonic suppression and operating bandwidth of the WPD. Different values of L_0_ and C_0_ combinations are considered for 0.8 GHz operating frequency in Table 3. Then the corresponding values of quality factors and operating bandwidths are extracted in Table 3.

### Layouts and electromagnetic (EM) simulation results for the third and fourth design examples

**Figure 11:**
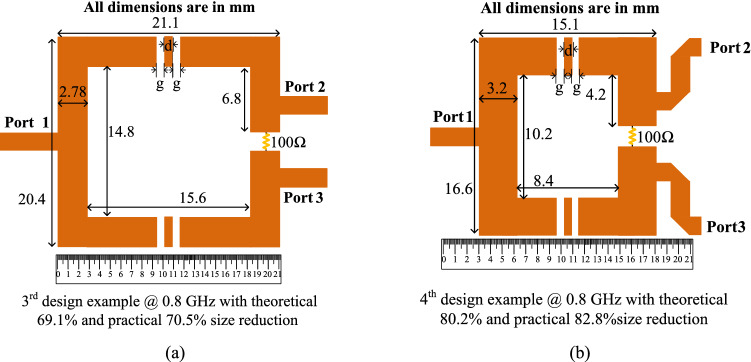
Layouts of the prototype dividers at 0.8 GHz for the (**a**) third design example with theoretical 69.1% size reduction and (**b**) fourth design example with theoretical 80.2% size reduction (All dimensions are in mm).

After selecting the desired size reduction and computing the circuit parameters for the third and fourth design examples, which are illustrated in the previous “Calculating the circuit parameters and circuit simulation results for the third and fourth design examples”, the layouts of these two design examples can be indicated. Same as the previous section, the used substrate for the layout of the design consists of an RT Duroid 5880 substrate with a thickness of 0.508 mm and ε_r_ = 2.2. The layouts of the prototype dividers at 0.8 GHz for the third and fourth design examples are shown in Fig. 11. On the basis of the information depicted in this Figure, the total size of the third and fourth design examples are 20.4 mm × 21.1 mm (0.075 λ_g_ × 0.077 λ_g_) and 16.6 mm × 15.1 mm (0.060 λ_g_ × 0.055 λ_g_), respectively. To calculate the practical size reduction of the designed examples, a layout of the conventional squared shaped WPD at 0.8 GHz is designed, in which the overall size of the conventional divider is obtained as 38.2 mm × 38.2 mm (0.14 λ_g_ × 0.14 λ_g_). Therefore, the practical size reduction of 70.5% and 82.8% are reached for the third and fourth design examples. In addition, in the third and fourth examples, the lengths of gaps (g) and intermediate transmission line between the gaps (d) are in order g = 0.6 mm and d = 0.5 mm.

**Figure 12:**
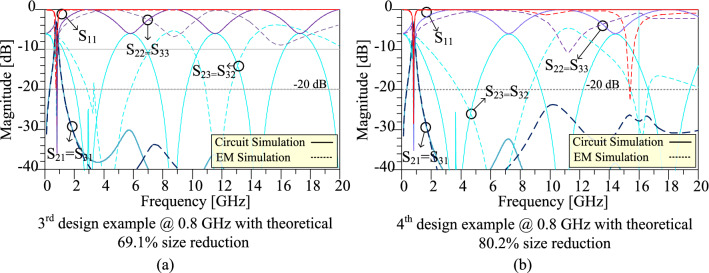
Frequency responses of the prototype WPDs at 0.8 GHz for the (**a**) third design example with theoretical 69.1% size reduction and (**b**) fourth design example with theoretical 80.2% size reduction.

The frequency responses of the prototype dividers at 0.8 GHz for the third and fourth design examples are depicted in Fig. 12. The circuit simulation and electromagnetic (EM) simulation are compared in this Figure. As it can be seen in this figure, the obtained EM simulation results verify the circuit simulation results. Based on the EM simulation results, for both third and fourth design examples, a suitable suppression band is obtained, which can suppress 2nd up to 25th spurious harmonics. In addition, for both third and fourth design examples, insertion loss is below 0.1 dB in the operating frequency, and isolation between output ports is better than 38 dB in the operating frequency. The detailed obtained EM simulation results for the third and fourth design examples are listed in Table 4 in “Measurement results”.

## Measurement results

**Figure 13:**
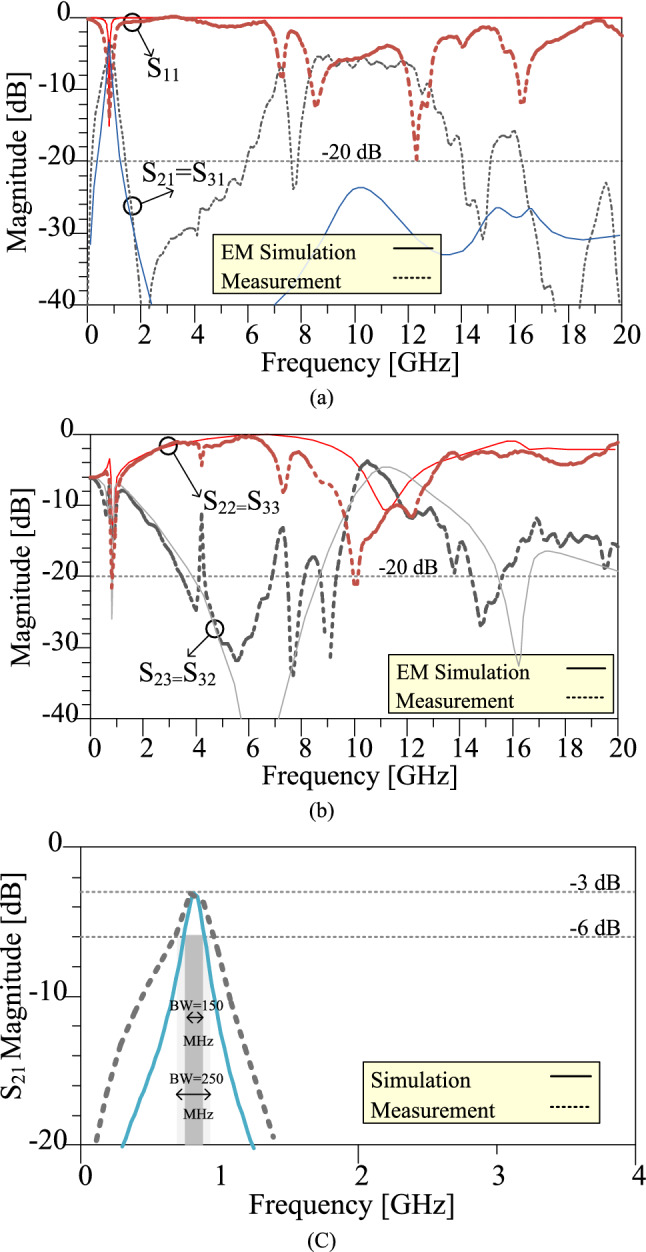
Frequency responses of the fabricated fourth design example at 0.8 GHz with theoretical 80.2% and practical 82.8% size reduction. (**a**) The S_21_ and S_11_ scattering parameters in the wide frequency range. (**b**) The S_32_ and S_22_ scattering parameters in the wide frequency range. (**c**) The S_21_ scattering parameter at about operating frequency range.

To verify the simulation and analyses results, the fourth design example with theoretical 80.2% and practical 82.8% size reduction at the operating frequency of 0.8 GHz is implemented on the high-frequency substrate with the specifications of RT Duroid 5880 with a thickness of 0.508 mm (20 mil) and ε_r_ = 2.2. Figure 13 compares experimental results with the EM simulation results. The fabricated divider is measured using Agilent E8362B Network Analyzer. The measurement is set up to 20 GHz frequency to clarify the harmonic suppression performance.

**Figure 14:**
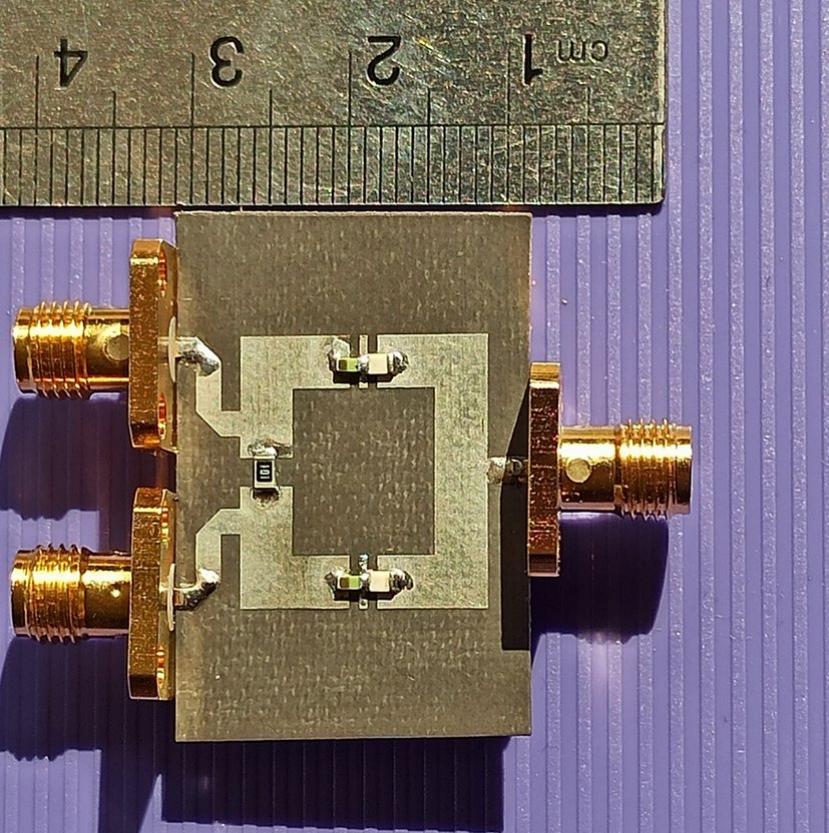
The fabricated proposed fourth design example of WPD at 0.8 GHz with theoretical 80.2% and practical 82.8% size reduction.

The measured results show that the proposed divider can practically operate at 0.7 GHz up to 0.95 GHz, which indicates 250 MHz operating bandwidth. The minimum insertion loss is 0.3 dB at this bandwidth. As is observed, the proposed divider can work properly at 0.8 GHz frequency with desirable specifications. Moreover, according to the measured results, the isolation between the output ports, input return loss, and output return loss is 15 dB, 14 dB and 22 dB, respectively. A proper suppression band is obtained for the proposed divider with a suppression level of more than 20 dB. The 2nd, 3rd, 4th, 5th, 6th, 7th, 8th, 10th, 17th, 18th, 19th, 20th, 21th, 22th, 23th, 24th, and 25th harmonics are suppressed with suppression levels of more than 15 dB, according to the measured results. The fabricated proposed WPD is shown in Fig. 14.

Table 4 provides information about the proposed divider results compared to the previous filtering dividers. Design Flexibility (DF) in this table means that the main parameters of the divider, such as size reduction, harmonic suppression, and bandwidth can be changed to the desired values. According to the information provided in Table 4, the proposed hybrid method is capable of designing a WPD with high design flexibility, low design complexity (DC), compact size, and high levels of harmonic suppression. Therefore, this innovative modification can contribute to considerable improvements in the design of the next generation of microwave components.

## Conclusions

An innovative hybrid technique for size reduction and performance improvement of a microstrip WPD has been conducted and demonstrated in this paper. In this method, microstrip lines in the WPD are replaced by the proposed LC branches. This replacement leads to extreme size reduction, filtering response, and improving performance. To assess the effectiveness of the proposed approach, four WPDs have been designed and simulated by amending their branch lines. This alteration in branches breeds an enormous size reduction and harmonic suppression in the dividers. In order to survey the practical performance of the designed WPDs, one of them was fabricated and its excremental results were measured. The results demonstrated the capability and efficiency of the introduced method to improve such components. Accordingly, 100% size reduction and an infinite number of harmonics suppression are reached theoretically. However, due to lumped element lengths, the theoretical size reduction can be mildly affected in practice, especially in high frequencies. In addition, folding and bending the microstrip lines may have slight effects on the theoretical values. Consequently, the proposed technique dramatically improves the efficiency and size of such components and provides new fields in the development of many other microstrip components, such as microstrip resonators, filters, diplexers, matching networks, couplers, power amplifiers, and the other types of dividers.

The following results can be concluded from the proposed method.

1. Arbitrary size reduction up to theoretically 100% can be achieved although the maximum size reduction percentage is limited to a number between 90 and 100% practically. The practicable size-reduction level also depends on the operating frequency and the lumped elements dimensions.
2. Infinite numbers of harmonic suppression can be observed in theory. However, the maximum number of harmonic suppression is limited up to about 20–30 harmonics practically. The practical numbers of harmonic suppression also depend on the operating frequency.
3. The operating bandwidth and sharpness of the divider can be tuned by setting the LC circuit values and different values of quality factors.
